# Standardization, Calibration, and Evaluation of Tantalum-Nano rGO-SnO_2_ Composite as a Possible Candidate Material in Humidity Sensors

**DOI:** 10.3390/s16122079

**Published:** 2016-12-07

**Authors:** Subbiah Karthick, Han-Seung Lee, Seung-Jun Kwon, Rethinam Natarajan, Velu Saraswathy

**Affiliations:** 1 Department of Architectural Engineering, Hanyang University, 1271 Sa 3-dong, Sangrok-gu, Ansan 426791, Korea; karthick.chemistry@gmail.com; 2 PG and Research Department of Chemistry, Alagappa Government Arts College, Karaikudi 630003, Tanilnadu, India; nataraajan12@yahoo.co.in; 3 Department of Civil Engineering, Hannam University, Daejeon 306-791, Korea; jjuni98@hannam.ac.kr; 4 Corrosion and Materials Protection Division, CSIR-Central Electrochemical Research Institute, Karaikudi 630003, Tamilnadu, India; corrsaras@gmail.com

**Keywords:** rGO-SnO_2_, nanocomposites, sensor, relative humidity

## Abstract

The present study focuses the development and the evaluation of humidity sensors based on reduced graphene oxide—tin oxide (rGO-SnO_2_) nanocomposites, synthesized by a simple redox reaction between GO and SnCl_2_. The physico-chemical characteristics of the nanocomposites were analyzed by XRD, TEM, FTIR, and Raman spectroscopy. The formation of SnO_2_ crystal phase was observed through XRD. The SnO_2_ crystal phase anchoring to the graphene sheet was confirmed through TEM images. For the preparation of the sensors, tantalum substrates were coated with the sensing material. The sensitivity of the fabricated sensor was studied by varying the relative humidity (RH) from 11% to 95% over a period of 30 days. The dependence of the impedance and of the capacitance with RH of the sensor was measured with varying frequency ranging from 1 kHz to 100 Hz. The long-term stability of the sensor was measured at 95% RH over a period of 30 days. The results proved that rGO-SnO_2_ nanocomposites are an ideal conducting material for humidity sensors due to their high sensitivity, rapid response and recovery times, as well as their good long-term stability.

## 1. Introduction

Humidity sensors play a vital role in environmental, industrial, and agricultural applications. Humidity is an important factor in physical, chemical, and biological processes. Optimal humidity is essential for human health and wellbeing. Humidity measurement in industrial processes is also critical because it may affect the quality and business cost of the product. Humidity sensors based on SnO_2_, TiO_2_, ZnO, CuO, and cross linked polymers have been reported in recent years [1,2,3,4,5,6,7,8,9,10]. Among them, SnO_2_ nanomaterials exhibit a higher performance for humidity sensing applications. It is due to their high surface area, high alkaline stability, excellent conductivity, good reversibility, ease of functionalization, robust mechanical properties, and low production cost. Moreover, the size and shape of the particles affect the humidity sensing capacity. Recently, high sensitivity humidity sensors based on a uniform morphology of SnO_2_ nanowires have been reported. The enhanced surface area and stable humidity sensing of the active material, which was prepared by adding GO, allowed the achievement of good performance in sensor applications [11,12]. In particular, numerous attempts have been made to combine reduced graphene oxide (rGO) with nano metal oxide to enhance their humidity sensing and photo catalytic properties, which are comparable with base materials. Seema et al., reported rGO-SnO_2_ nanocomposites synthesized by a simple chemical method, that they exhibited enhanced photo catalytic activity regarding the degradation of methylene blue under sunlight [13]. Zhang et al., demonstrated the good performance of NO_2_ sensing at low temperatures by SnO_2_ nanoparticles decorated with rGO nanocomposite [14]. Morimutu et al., reported on surface modification of platinum and ruthenium over SnO_2_ particles for CO gas sensors [15]. However, to the best of our knowledge, there have been few reports that have focused on SnO_2_ nanoparticle humidity sensors anchored to a graphene sheet.

Most of the studies deal with highly conductive materials used in ceramic substrate coated with noble metals such as Au, Pt, Pd, and Ag. This process can be achieved by physical or chemical methods. These methods tend to be interactive in a number of cases due to random operation cost effects. Alternative cost-effective materials like tantalum exhibit an excellent dielectric constant, high capacitive behavior, and enhanced corrosion resistance to moisture and aqueous solutions [16]. Hence, tantalum is an ideal candidate for the base substrate material for humidity sensor applications.

In the present study, for the first time, attempts were made to attach SnO_2_ on a graphene sheet for humidity sensing application. This investigation lead to a novel humidity sensor of nano-rGO-SnO_2_ over a tantalum support.

## 2. Materials and Methods

### 2.1. Materials

All used chemicals were of analytical grade and utilized without further purification. Tin chloride dihydrate (SnCl_2_·2H_2_O), Hydrochloric acid (HCl) and urea were purchased from SRL Chem. Graphite powder, PVDF (Poly (vinylidene fluoride)), carbon black, and NMP (1-*N*-methyl-2-pyrrolidinone) were purchased from Alfa Aesar. Tantalum was purchased from Manhar Metal supply Corporation, Mumbai, in India.

### 2.2. Preparation of Nano SnO_2_ and rGO-SnO_2_ Composites

SnCl_2_·2H_2_O (1.30 g) was dissolved in HCl (38 wt %, 0.7 mL) and diluted with distilled water (20 mL) to give a SnCl_2_:HCl solution. In order to form a uniform solution, urea (1.30 g) was added to the SnCl_2_:HCl solution under vigorous stirring. The resulting solution was then heated up and kept at 90 °C for 12 h. The obtained precipitate was separated by centrifugation and washed with water to remove excess chloride ions. The resulting product was dried in a vacuum oven at 110 °C for 24 h.

GO was prepared from powdered flake graphite (400 mesh) by a modified Hummers method as described elsewhere [17,18]. The obtained graphite oxide (10 mg) was dispersed in distilled water (20 mL) using sonication to form a colloidal suspension. The GO solution was added to a SnCl_2_:HCl solution: then urea was added to the previously obtained solution and heated up and kept at 90 °C for 12 h. The resulting precipitate was separated by centrifugation and subsequent washing, to remove excess chloride ions. The resulting product was dried in a vacuum oven at 110 °C for 24 h.

### 2.3. Characterization Techniques

The obtained SnO_2_ and rGO-SnO_2_ were analyzed by FTIR spectroscopy. The sample was mixed with KBr crystal and the spectrum was recorded in the transmittance mode in the range of 400–4000 cm^−1^.

The phase structure of the nanocomposite was characterized by XRD. A computer controlled XRD system—comprised of JEOL, JPX-8030 with Cu-Kα radiation (Ni filter = 13,418 Å), and a power source with 40 kV and 20 A—was used to record the diffraction patterns. Identification of diffraction peaks was done by a computer controlled software application (PAN analytical, X’pert High score plus).

The surface defects, D and G peak, sp^2^ hybridization of GO, SnO_2_, and rGO-SnO_2_ were determined by Laser-Raman spectroscopy (Renishaw Invia Raman Microscope with Wire-2 software) with an excitation wavelength of 633 nm and an exposure time of 50 s (100% intensity).

To visualize the nanocomposites, the particles were dispersed in water, sonicated for an hour and casted onto a copper grid to study the sizes and morphology of the particles on the GO sheet by TEM (Transmission Electron Microscopy) using Tecnai 20G2-FEI, with an acceleration voltage of 200 kV.

### 2.4. Assembly and Measurement of Humidity Sensors

**Figure 1:**
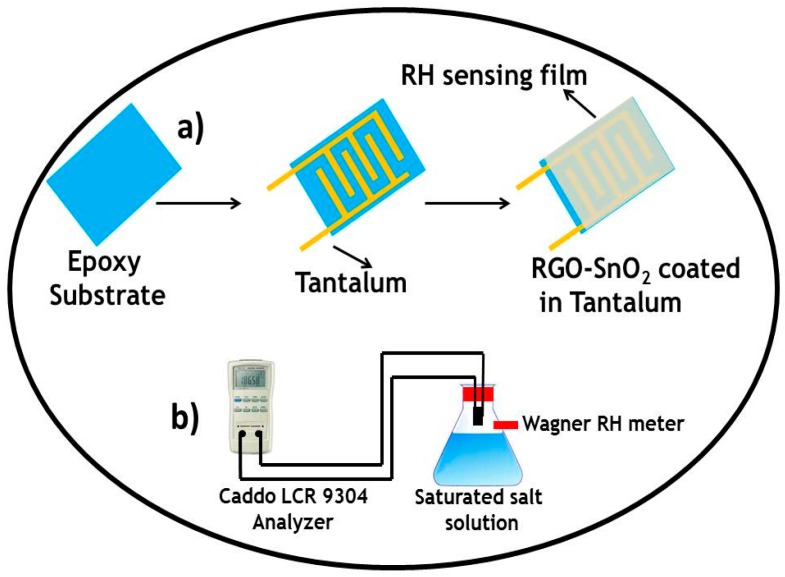
Schematic diagram of (**a**) the Humidity sensor; (**b**) the Measurement system.

Devices with interdigital electrodes were used for humidity sensing measurement. The interdigital electrodes were fabricated by using a tantalum sheet of 1 mm thickness, applying an epoxy substrate, and coating it with rGO-SnO_2_. As shown in Figure 1a, the width of a finger was about 0.1 mm, and the distance between adjacent fingers around 75 mm. Two lead wires were connected to the tantalum electrodes by tin soldering, followed by masking with epoxy resin. Then, a thick slurry of rGO-SnO_2_ (90%), carbon black (5%), and Poly vinylidene fluoride (PVDF) (5%) in *N*-methyl-2-pyrrolidinone (NMP) solvent was prepared and coated on top of the interdigital electrodes (each electrode has six arms) previously treated with an epoxy substrate by the doctor blade technique and dried at 80 °C in air for 12 h in order to evaporate the solvent. Finally, the humidity sensor was fabricated and aged under a relative humidity (RH) of 95%, and with a sinusoidal voltage with an amplitude of 1 V, at 100 Hz, for 24 h. Figure 1a shows a schematic diagram of the humidity sensor. The total thickness of the sensor is 3 mm distributed as follows: epoxy substrate, 2 mm; tantalum, 0.5 mm; and rGO-SnO_2_ coating, 0.5 mm. The characteristic humidity sensitivity curves were measured on a Caddo LCR-9304 analyzer. For that purpose, a sinusoidal voltage with an amplitude of 1 V, whose frequency varied from 100 Hz to 1 kHz at a temperature of 20 °C, was used. The relative atmospheric humidity was produced using different saturated salt solutions in their equilibrium states, including LiCl for 11% RH, MgCl_2_ for 33% RH, NaCl for 75% RH, KCl for 85% RH, and K_2_SO_4_ for 95% RH at about 20 °C [19]: the uncertainty of the RH values was about ±1%. The humidity sensing measurement system is shown in Figure 1b.

## 3. Results and Discussion

### 3.1. Formation Mechanism of SnO_2_ and rGO-SnO_2_ Nanocomposites

SnO_2_ nanoparticle decorated graphene sheets were synthesized via a redox reaction between graphene oxide and SnCl_2_·2H_2_O. An aqueous dispersion of GO was mixed with an SnCl_2_:HCl solution. During the redox reaction, the anchored Sn^2+^ cations were oxidized to SnO_2_, while the graphene oxide sheets were reduced to graphene sheets. The even loading of SnO_2_ nanoparticles on both sides of the graphene layer prevented the aggregation of the graphene layers. The possible reaction mechanism can be written as follows [20]:

SnCl_2_ + GO + H_2_O → rGO-SnO_2_ + 2HCl (1)

CO(NH_2_)_2_ + H_2_O → CO_2_ + 2NH_3_ (2)

NH_3_ + H_2_O → NH_4_OH (3)

NH_4_OH + HCl → NH_4_Cl + H_2_O (4)

### 3.2. Characterization of Nano SnO_2_ and rGO-SnO_2_ Nanocomposites

#### 3.2.1. Fourier Transform Infrared Spectroscopy (FTIR)

**Figure 2:**
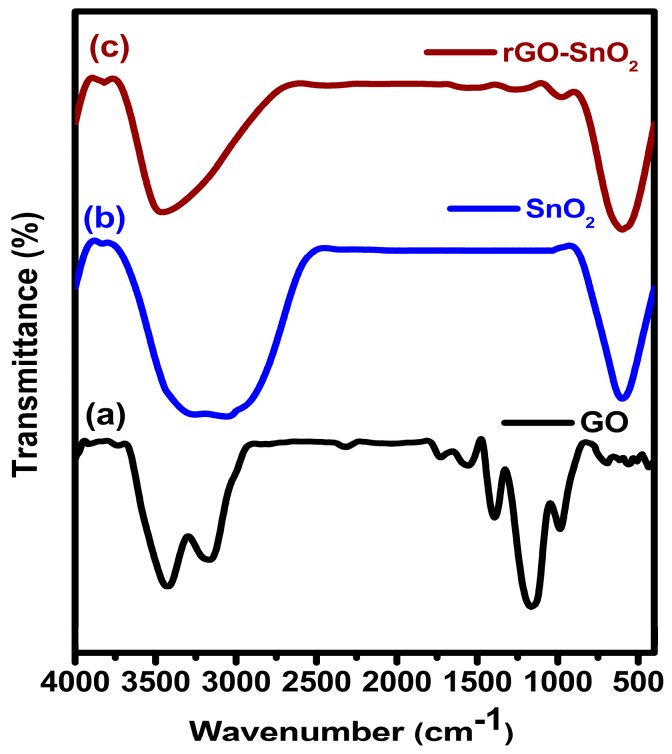
FTIR spectra for (**a**) GO; (**b**) SnO_2_; (**c**) rGO-SnO_2_.

The GO, SnO_2_, and rGO-SnO_2_ were recorded in the FT-IR spectra at transmittance mode and are shown in Figure 2a–c, respectively. The peaks observed at 3453 cm^−1^ and 1400 cm^−1^, correspond to the O–H stretching of –COOH in GO and to the vibration of intercalated water, respectively; the characteristic peaks of oxygen moieties—located at 1184 cm^−1^, 1595 cm^−1^, and 1747 cm^−1^—correspond to C–O (*ν* (epoxy or alkoxy)), C=O in –COOH, and carbonyl moieties (*ν* (carbonyl), respectively. The GO functional groups are significantly reduced from GO-SnO_2_ materials. It is clearly indicated that the GO is partially reduced to graphene. Moreover, the new peak (Figure 2b) appeared at 597 cm^−1^, is attributed to the Sn–O stretching. From this spectrum, it is clear that the rGO-SnO_2_ peak intensities are similar to the ones of the SnO_2_ sample. The above facts enunciate the possible compact chemical bonding between SnO_2_ and rGO, which may be due to the unique synthetic approach.

#### 3.2.2. X-ray Diffraction (XRD)

**Figure 3:**
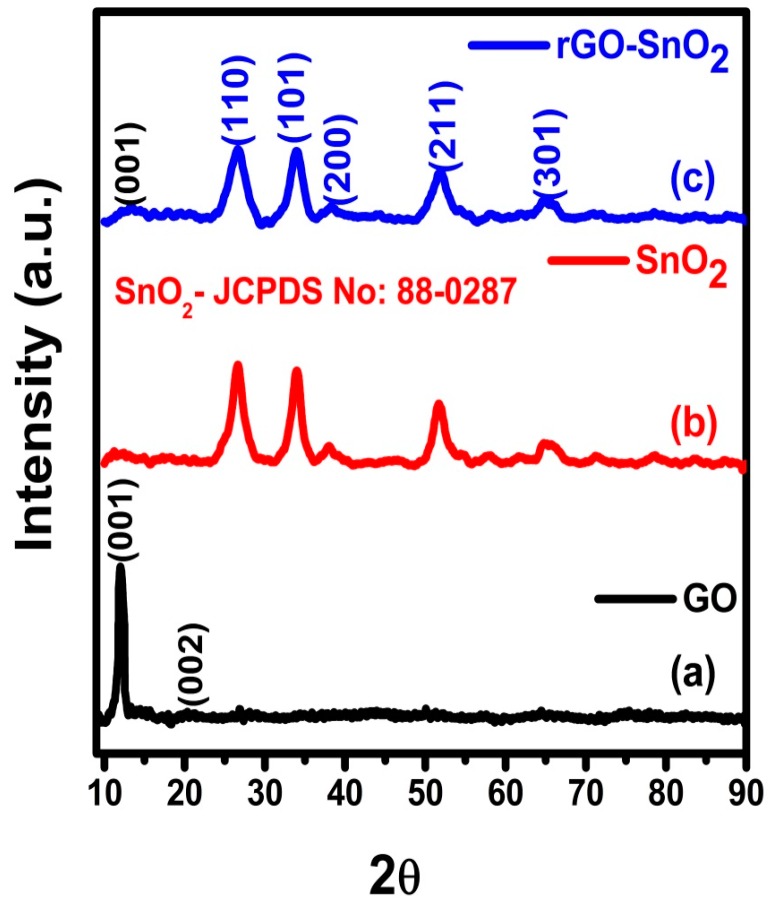
XRD pattern for (**a**) GO; (**b**) SnO_2_; (**c**) rGO-SnO_2_.

Figure 3a–c shows the XRD pattern for GO (a), SnO_2_ (b), and rGO-SnO_2_ (c), respectively. Figure 3a shows the diffraction peaks at 11.9° corresponding to the exfoliation of graphitic plane, i.e., (001) crystal plane structure [21]. In Figure 3b, the diffraction peaks observed at 26.7°, 34.2°, 37.9°, 51.9° and 65.7° correspond to SnO_2_ (110), (101), (200), (211), and (301) crystal phases, respectively. The diffraction peaks of the synthesized rGO-SnO_2_ match the ones of the tetragonal crystal phase of SnO_2_ (ICDD NO: 88-0287). In Figure 3c, the observable diffraction peaks were compared with the patterns of GO and SnO_2_. Furthermore, the reduced intensity of the diffraction peak corresponding to the graphitic plane confirms the reduction of GO (11.9°) [22]. The mean particle size (D) of the SnO_2_ nanoparticles was calculated using the Scherrer formula: by applying it to the (110) plane diffraction peak, it was found that D is ~30 nm for the rGO-SnO_2_ composite and ~50 nm for the SnO_2_ nanoparticles alone.

#### 3.2.3. Raman Spectra

**Figure 4:**
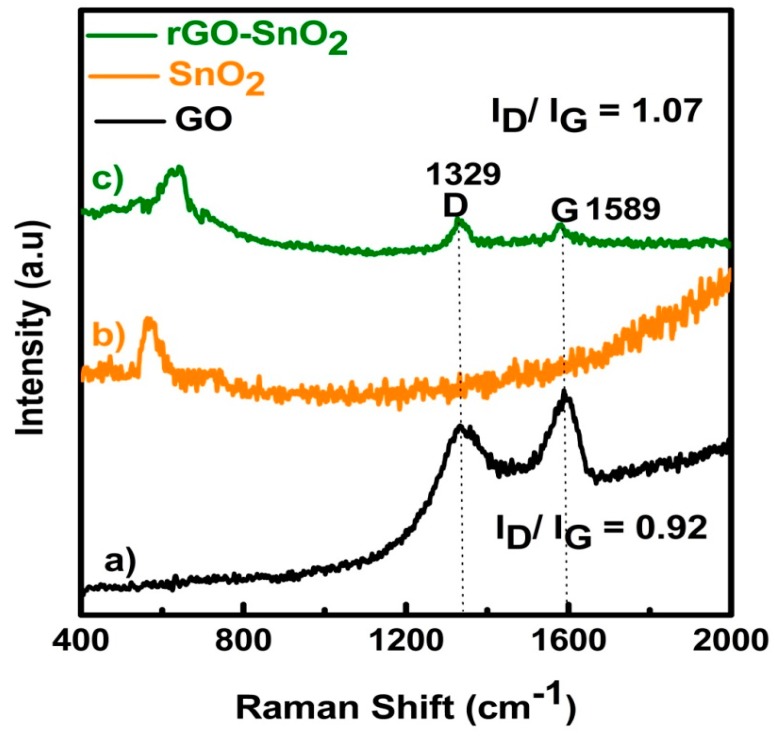
Raman spectra for (**a**) GO; (**b**) SnO_2_; (**c**) rGO-SnO_2_.

In Figure 4, the Raman spectra of GO (a), SnO_2_ (b), and rGO-SnO_2_ (c) are plotted respectively. The observed Raman peak shift at 576 and 630 cm^−1^, in Figure 4b,c, respectively, indicates the presence of SnO_2_ [23]. Additionally, in the rGO-SnO_2_ spectrum, the peaks at 1329 and 1589 cm^−1^ correspond to the D and G band of GO. Previous reports have shown that the D band always appears whether any form of disorders and defects are present; e.g., vacancies, grain boundaries, and amorphous carbon species in carbon materials. The G band is usually generated by the stretching of all sp^2^ atoms in carbon rings or carbon chains (E_2g_ photons of sp^2^ atoms). Namely, the I_D_/I_G_ intensity ratio is closely related with the concentration of defects and with the average size of the p-conjugation in carbon materials. Reasonably, the D band will disappear gradually when the GO is reduced for defect decrease. In the present work, the I_D_/I_G_ ratios are 0.92 for GO and 1.07 for rGO-SnO_2_ [24], respectively. Therefore, the existence of SnO_2_ in rGO results in more defects, as well as in the destruction of sp^2^ domains. From another point of view, this change also suggests an interesting connection between the SnO_2_ nanoparticles (NPs) and rGO [25].

#### 3.2.4. Transmission Electron Microscope (TEM)

**Figure 5:**
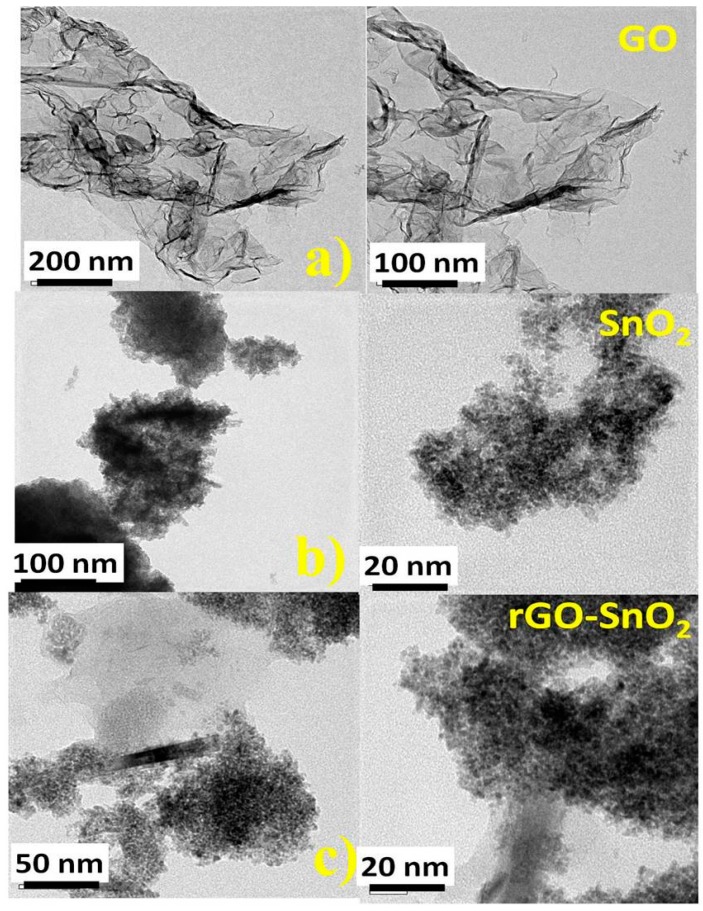
TEM image for (**a**) GO; (**b**) SnO_2_; (**c**) rGO-SnO_2_.

TEM images of GO, snO_2_, and rGO-SnO_2_ nanocomposites are shown in Figure 5a–c respectively. Figure 5a shows the TEM images of ultrasonically exfoliated graphene oxide. It clearly indicates the typical attributes of GO such as wrinkles and crumpled thin paper-like structure, with numerous folds at the edges of the GO sheet [26]. This feature has been reported previously by various researchers. As expected, the TEM images illustrate that GO was exfoliated into very thin layers. Figure 5b shows the TEM image of the SnO_2_ nanoparticles. Interestingly, the structure is composed of quasi-spherical nanoparticles. The size distribution and particle diameter were estimated using the ImageJ software application: it was found to lie in the range between 40 nm and 50 nm. A similar SnO_2_ lattice fringe was also observed for the rGO-SnO_2_ nanocomposites (Figure 5c). The TEM studies further confirmed the incorporation of SnO_2_ nanoparticles on the surface of rGO [27].

### 3.3. Humidity Sensing Properties of rGO-SnO_2_ Sensor

**Figure 6:**
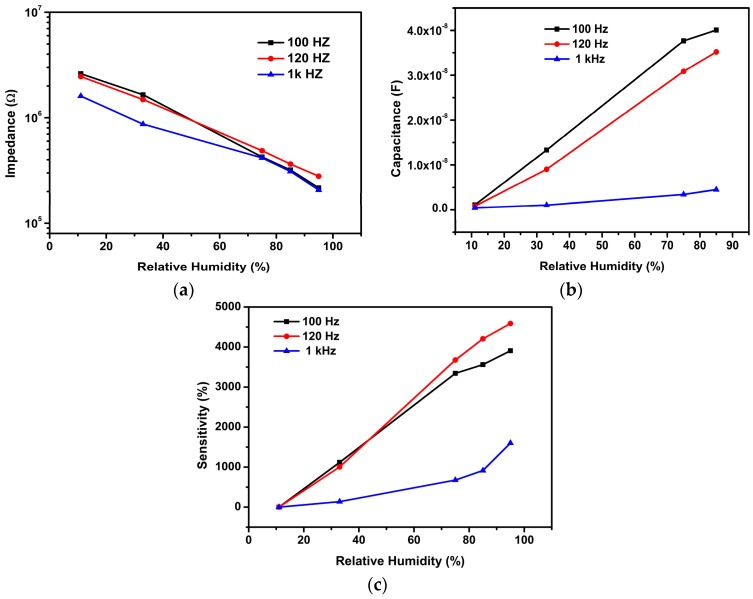
(**a**) The dependence of impedance on the RH for the rGO-SnO_2_ sensor measured at various frequencies; (**b**) The dependence of capacitance on the RH for the rGO-SnO_2_ sensor measured at various frequencies; (**c**) The sensitivity of the rGO-SnO_2_ sensor for different tested RH and frequencies.

To determine the optimal working parameters, the impedance of the rGO-SnO_2_ sensor under different RH atmospheres and for different frequencies (100, 120, and 1 kHz) was measured at 20 °C, and the results are shown in Figure 6a. The impedance of the sensor under certain RH decreased with increasing working frequency. It is worth to noting that the impedance variation at low RH is much bigger than that observed at high RH. The sensing curves almost overlap for RH higher than 80%, indicating that the effect of frequencies on impedance was very small for this range. On the other hand, the impedance decreased with the increasing RH, due to the increase of absorbed water molecules onto the sensitive layer [28]. It can be seen that the impedance of the sensor strongly depends on the measuring frequency and the RH level. A large change is observable for the lower tested frequencies of 100 Hz and 120 Hz, while the change is inconspicuous for the higher tested frequency of 1 kHz as reported by other authors [29]. This is because the electrical field direction changes slowly at low frequencies and so polarization of the adsorbed water is strong. However, for the higher frequency ranges, the electrical field direction changes fast, and consequently water polarization cannot catch up with it: in consequence, the dielectric constant is small and does not change with RH [30,31]. The sensing curve at 120 Hz exhibits the best linearity, and at this frequency the sensor is very sensitive for the whole humidity range, with the impedance changing four orders of magnitude when RH varied from 11% to 95% RH. So the optimum measurement conditions were fixed for the remaining experiments: sinusoidal voltage with amplitude of 1 V, and a frequency of 120 Hz. Figure 6b shows the dependence of the sensor capacitance on RH at different frequencies (100 Hz, 120 Hz, and 1 kHz). The reported data are the mean values obtained from several measurement cycles at a temperature of 20 °C. As the RH level increases, the output capacitance of the sensor shifts higher monotonically as observed by other authors [32]. Adsorbed water can increase the dielectric constant and the capacitance. Furthermore, adsorbed water molecules strengthen the polarization and increase the dielectric constant [33]. The sensitivity of the sensors, in percentage, at different RHs was calculated using the following equation [34].

(5) $$S=\frac{C_{RH}-C_{11}}{C_{11}}\times 100$$

where *C*_11_ and *C_RH_* stand for the capacitances measured at 11% RH and at a certain RH level, respectively. The calculated sensitivity of the sensor across the RH range of 11–95% was ~3900%, ~4600%, and ~1581% for the excitation signal frequencies of 100 Hz, 120 Hz, and 1 kHz respectively The sensitivity of our sensor at the frequency of 120 Hz is higher than that found for the frequencies of 100 Hz and 1 kHz [35]. Therefore, in the remainder of the study, the frequency of 120 Hz was considered the best test frequency for the analysis of sensor characteristics.

### 3.4. Humidity Sensors’ Response-Recovery Time

The response and recovery times are important parameters to estimate the effectiveness of humidity sensors. The measurement method was as follows. First, the sensor was put into a glass container at 11% RH for several minutes until the impedance of the sensor became steady. Then, the sensor was transferred into a glass vessel at 95% RH as quickly as possible (less than 2 s) in order to reduce the effect of the laboratory atmosphere. Finally, the sensor was moved back to the chamber containing 11% RH, once the sensor impedance stabilized at 95% RH [36].

**Figure 7:**
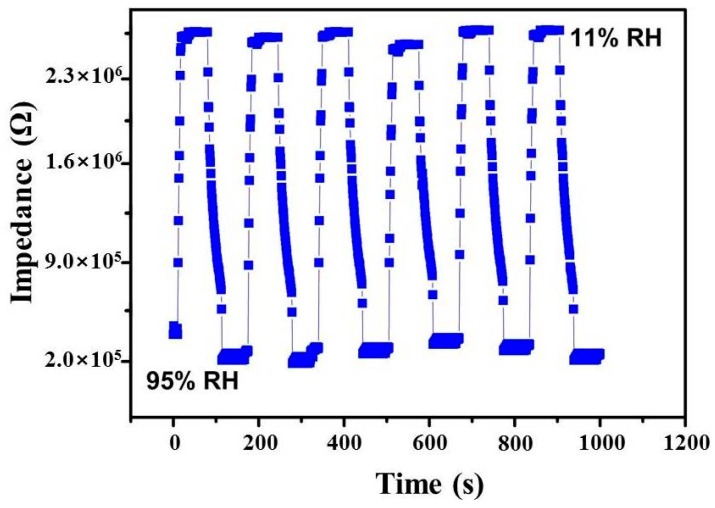
Response and recovery curves of the rGO-SnO_2_ sensor.

The impedance modulus variation of the rGO-SnO_2_ sensor for four consecutive cycles of the above described process of water adsorption and desorption is shown in Figure 7. The observed response time is 10 s, while the recovery time is 60 s. The response and recovery properties are consistent with the low humidity hysteresis exhibited by the sensor [32,37]. Comparison of response and recovery times between the proposed sensor and other sensors reported in the literature is given in Table 1.

Li et al. [38] have studied the structure and humidity sensing properties of SnO_2_ zigzag belts and the calculated response and recovery times based on different RHs (5%, 22%, 43%, 69%, and 97%) were in the intervals 30–110 s and 80–150 s, respectively. Similarly, Qin Kuang et al. [39] have prepared a high-sensitivity humidity sensor based on a single SnO_2_ nanowire: the calculated response and recovery times, based on different RHs (5%, 30%, 48%, 76%, and 85%), were in the ranges 120–170 s and 20–60 s, respectively. Sin et al. [40] have prepared a humidity sensor based on a film of nanocubic ZnO:SnO_2_ with high sensitivity, by ultrasonic-assisted solution growth method with different Zn:Sn precursor ratios. The calculated response and recovery times were of 411 s and 98 s, respectively. Compared to the above values, the rGO-SnO_2_ nanoparticles have a better sensitivity factor and faster response and recovery times. The fast response of the sensor is also attributed to the large quantity of hydrophilic quaternary SnO_2_ groups in the rGO [47], which interact quickly with water molecules, while the rapid recovery speed is due not only to the separation of sensitive points in the framework, but also to the porosity, which is characteristic of rGO-SnO_2_ [48,49].

**Figure 8:**
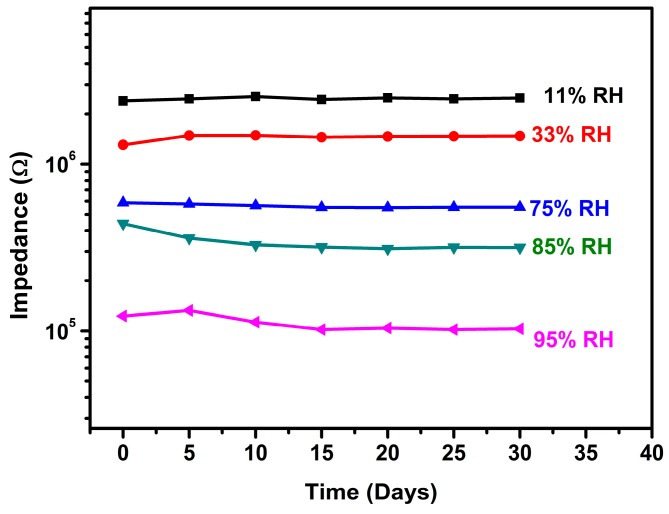
The long-term stability of the rGO-SnO_2_ sensor after being exposed to 95% RH for 30 days.

To test the long-term stability at high humidity, the rGO-SnO_2_ sensor was placed in the glass container with 95% RH and the impedance measurements were repeated every five days over a period of 30 days. As shown in Figure 8, a slight variation in impedance was observed with the passage of time, proving that the rGO-SnO_2_ sensor has good stability and durability at high humidity. This is highly probably due to the cross-linked porous framework of the rGO-SnO_2_ [50], which could effectively prevent the composites from dissolving in the adsorbed water and immobilize the functional molecules (rGO) in the pores.

### 3.5. Sensing Mechanism of rGO-SnO_2_

**Figure 9:**
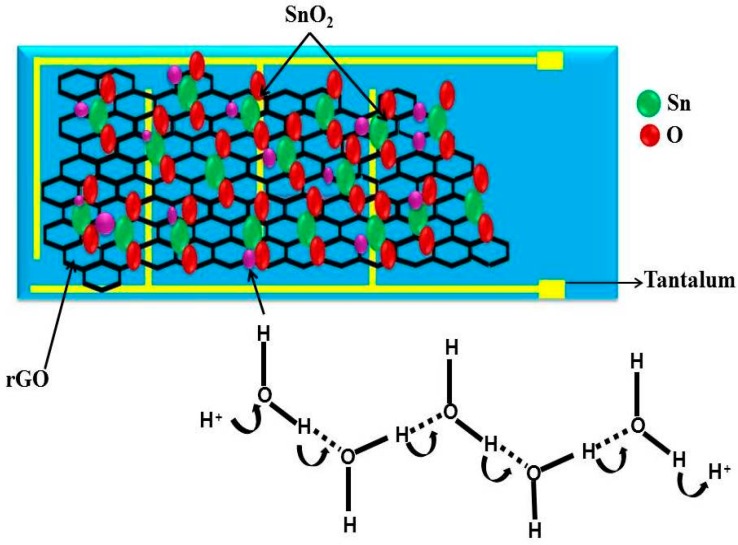
The schematic of the proposed humidity sensing mechanism of the rGO-SnO_2_ nanocomposite.

**Figure 10:**
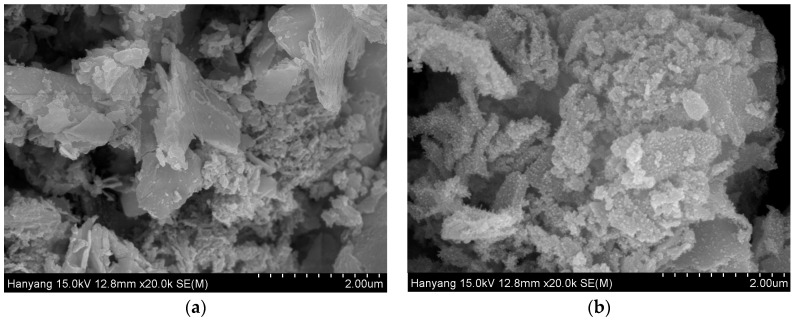
SEM microstructure image for (**a**) SnO_2_; (**b**) rGO-SnO_2_.

The sensing mechanism is based on humidity adsorption and desorption process on the surface structure [51,52]. The sensing adsorption occurs in SnO_2_ by its conduction inside the water layers and is shown in Figure 9. When a water molecule comes in contact with the surface of the SnO_2_, hydroxyl ions combine with Sn. The ionized oxygen combined with proton form a surface layer on top of the SnO_2_ particles [53]. At this stage, water molecules get condensed and cannot move freely due to the two hydrogen bonding. The first physically adsorbed layer has a higher order than the upcoming adsorbed layer. Continuous condensing of water breaks the single hydrogen bonding; protons start to move freely [54]. SnO_2_ nanocrystals have an effective electronic interaction with rGO [55,56,57], hence the rGO-SnO_2_ facilitates the detection of water molecule. The rGO-SnO_2_ system can detect the water molecules that are normally undetectable by rGO, since the rGO-SnO_2_ system is potentially superior. rGO-SnO_2_ exhibits higher sensitivity than pure rGO, since SnO_2_ nanoparticles combined with rGO offer more active sites such as vacancies, defects, and oxygen functional groups, as does the sp^2^ bonded carbon regarding the adsorption of water molecules; also due to the high surface area and electrical conductivity. The observed microstructure of SnO_2_ and rGO-SnO_2_ is shown in Figure 10. SnO_2_ microstructure has a smooth surface (Figure 10a) and is more hydrophilic in nature [47]. This hydrophilic property of SnO_2_ always enhances the fast sensing response towards the water molecules, but decreases the recovery time. This is due to the condensed water molecules on the surface of SnO_2_ that cannot move freely due to the hydrogen bonding [54]. After the anchoring of SnO_2_ on the rGO sheet, it increases the surface roughness (Figure 10b). Hence, it decreases the physical adsorption between water molecules and the sensing material, which enhances the recovery time.

## 4. Conclusions

rGO-SnO_2_ nanocomposites were synthesized via redox reaction and used for humidity sensing application. The humidity sensor was fabricated using rGO-SnO_2_ on tantalum substrate to access the sensing platform. The sensitivity of the fabricated sensor was studied by varying the relative humidity (RH) from 11% to 95% over a period of 30 days. The impedance and capacitance variation on RH as well as the long-term stability of the sensor measured at 95% RH over a period of 30 days, proving that rGO-SnO_2_ nanocomposite exhibited high sensitivity, rapid response and recovery times, and good long-term stability.

## Figures and Tables

**Table 1:** Comparison of the proposed sensor with other reported sensors in the literature.

Reference	Electrode	Substrate	Sensing Material	Sensor Response Time (s)	Sensor Recovery Time (s)
Bi et al. [32]	Gold (Au)	SiO_2_	Graphene oxide	10.5	41
Li et al. [38]	Sliver (Ag)	SiO_2_	SnO_2_	30–110	80–150
Kuang et al. [39]	Platinum (Pt)	SiO_2_	SnO_2_	120–170	20–60
Sin et al. [40]	Gold (Au)	SiO2	ZnO/SnO_2_	411	98
Zhang et al. [41]	Platinum (Pt)	SiO_2_	ZnO	10	30
Chen [42]	Copper (Cu)	SiO_2_	MWCNTs	45	15
Chen et al. [43]	Silver (Ag)	Si	SiNWs	350	52
Chen et al. [43]	Silver (Ag)	Si	HMDS modified SiNWs	132	62
Wang et al. [44]	Porous Silicon	Si	Ta_2_O_5_	18	40
Wang et al. [45]	Silver (Ag)	Ceramic	TiO_2_	5	10
Sun et al. [46]	Gold electrode	Aluminum	Polypyrrole	41	120
Proposed differentiate	Tantalum electrode	Epoxy	rGO-SnO_2_	10	60
